# Imaging Spectrum of the Developing Glioblastoma: A Cross-Sectional Observation Study

**DOI:** 10.3390/curroncol30070490

**Published:** 2023-07-13

**Authors:** Stuart Currie, Kavi Fatania, Russell Frood, Ruth Whitehead, Joanna Start, Ming-Te Lee, Benjamin McDonald, Kate Rankeillor, Paul Roberts, Aruna Chakrabarty, Ryan K. Mathew, Louise Murray, Susan Short, Andrew Scarsbrook

**Affiliations:** 1 Department of Neuroradiology, Leeds Teaching Hospitals NHS Trust, Leeds General Infirmary, Floor B, Clarendon Wing, Great George Street, Leeds LS1 3EX, UK; 2 Leeds Institute of Medical Research, University of Leeds, Leeds LS2 9TJ, UK; louise.murray8@nhs.net (L.M.); susan.short4@nhs.net (S.S.); a.scarsbrook@nhs.net (A.S.); 3 Radiology Academy, Leeds Teaching Hospitals NHS Trust, Leeds General Infirmary, Floor B, Clarendon Wing, Great George Street, Leeds LS1 3EX, UK; kavi.fatania@nhs.net (K.F.); russellfrood@nhs.net (R.F.); ruth.gilliver1@nhs.net (R.W.); joanna.start@nhs.net (J.S.); ming-te.lee@nhs.net (M.-T.L.); 4 Department of Histopathology, Leeds Teaching Hospitals NHS Trust, St James’s University Hospital, Leeds LS9 7TF, UK; benjamin.mcdonald@nhs.net (B.M.); kate.rankeillor@nhs.net (K.R.); paul.roberts16@nhs.net (P.R.); arundhati.chakrabarty@nhs.net (A.C.); 5 Department of Neurosurgery, Leeds Teaching Hospitals NHS Trust, Leeds General Infirmary, Floor G, Jubilee Wing, Great George Street, Leeds LS1 3EX, UK; 6 School of Medicine, University of Leeds, Leeds LS2 9JT, UK; 7 Department of Clinical Oncology, Leeds Teaching Hospitals NHS Trust, St James’s University Hospital, Leeds LS9 7TF, UK; 8 Department of Radiology, Nuclear Medicine, Leeds Teaching Hospitals NHS Trust, Bexley Wing, St James’s University Hospital, Leeds LS9 7TF, UK

**Keywords:** brain, glioblastoma, computed tomography, magnetic resonance imaging

## Abstract

Glioblastoma (GBM) has the typical radiological appearance (TRA) of a centrally necrotic, peripherally enhancing tumor with surrounding edema. The objective of this study was to determine whether the developing GBM displays a spectrum of imaging changes detectable on routine clinical imaging prior to TRA GBM. Patients with pre-operative imaging diagnosed with GBM (1 January 2014–31 March 2022) were identified from a neuroscience center. The imaging was reviewed by an experienced neuroradiologist. Imaging patterns preceding TRA GBM were analyzed. A total of 76 out of 555 (14%) patients had imaging preceding TRA GBM, 57 had solitary lesions, and 19 had multiple lesions (total = 84 lesions). Here, 83% of the lesions had cortical or cortical/subcortical locations. The earliest imaging features for 84 lesions were T2 hyperintensity/CT low density (*n* = 18), CT hyperdensity (*n* = 51), and T2 iso-intensity (*n* = 15). Lesions initially showing T2 hyperintensity/CT low density later showed T2 iso-intensity. When CT and MRI were available, all CT hyperdense lesions showed T2 iso-intensity, reduced diffusivity, and the following enhancement patterns: nodular 35%, solid 29%, none 26%, and patchy peripheral 10%. The mean time to develop TRA GBM from T2 hyperintensity was 140 days and from CT hyperdensity was 69 days. This research suggests that the developing GBM shows a spectrum of imaging features, progressing through T2 hyperintensity to CT hyperdensity, T2 iso-intensity, reduced diffusivity, and variable enhancement to TRA GBM. Red flags for non-TRA GBM lesions are cortical/subcortical CT hyperdense/T2 iso-intense/low ADC. Future research correlating this imaging spectrum with pathophysiology may provide insight into GBM growth patterns.

## 1. Introduction

Glioblastoma (GBM) is the most common malignant adult brain tumor. It carries a poor prognosis, with a mean survival between 14 and 16 months for patients treated with the standard strategies (maximal safe surgical resection, followed by radiotherapy together with concomitant and adjuvant chemotherapy using temozolomide (TMZ)—the so-called Stupp regime [1,2]). The 2021 WHO Classification [3] describes GBM as an adult-type WHO Grade 4 isocitrate dehydrogenase wildtype (IDHwt) glioma with the typical histological features of mitoses, vascular endothelial proliferation, and necrosis. A diagnosis of GBM can also be made in the absence of typical histological features if one of the following three molecular alterations is present: (1) epidermal growth factor (EGFR) gene amplification; (2) chromosome 7 polysomy ± chromosome 10 monosomy; and (3) telomerase reverse transcriptase (TERT) promotor mutation [4]. Patients with GBM stereotypically present with a significant disease burden. The typical radiological appearance of GBM (TRA GBM) at the time of diagnosis is a centrally necrotic, peripherally enhancing cerebral tumor surrounded by an infiltrative high signal on T2-weighted and fluid-attenuated inversion recovery (FLAIR) imaging, with the latter high signal reflecting an admixture of infiltrative tumor and vasogenic edema [4]. GBM are relatively large tumors [5], and volumetric GBM imaging features are associated with overall survival (OS) [6]. Detection of GBM at an earlier stage may improve the prognosis.

Early imaging features during the development of GBM are seldom captured, presumably due to minimal symptomatic manifestations [7]. Some authors have reported small MR imaging lesions that subsequently progress to GBM [8,9,10,11,12,13,14,15,16,17,18,19], often described as ill-defined FLAIR or T2-weighted hyperintensities without mass effect that typically involves both the cortex and subcortical white matter but occasionally appears as only cortical lesions [9,11,15]. CT-hyperdense lesions have also been reported [20]. Contrast enhancement is an inconsistent feature but tends to be focal and nodular when present [13,14,15]. It remains possible that all GBMs, irrespective of their location of origin, move through a spectrum of imaging changes that incorporate some of the imaging features previously reported in the literature. Those reported to date gain attention because the predominantly cortical location triggers symptoms that demand a medical investigation.

The purpose of this study was to investigate whether the developing GBM displays a spectrum of imaging changes that are detectable on routine clinical imaging. The presence of such a spectrum may have implications for GBM detection, treatment, and monitoring.

## 2. Materials and Methods

This observational cohort study had ethical approval (IRAS ref: 277122, Enhancing understanding and prediction of cancer outcomes with baseline characteristics from routinely collected data), and patient informed consent was obtained.

### 2.1. Patient Selection and Data Collection

Neuro-oncology multidisciplinary team (MDT) meeting records at a tertiary neuroscience UK NHS Trust between 1 January 2014 and 31 March 2022 were retrospectively reviewed, and all adult patients (16 years and over) with IDHwt GBM WHO Grade 4 tumors were included. Our institution serves a catchment area of 3.9–4.4 million adults.

Patient electronic health records were accessed using in-house software. Baseline data included age, sex, presenting complaint, and operation type (resection or biopsy). OS was calculated as the interval between the first operation and the patient’s death.

Histopathological and genomic data included histology, IDH 1 and 2 mutations, O6-methylguanine-DNA methyltransferase (MGMT) promoter methylation, 1p19q co-deletion, v-Raf murine sarcoma viral oncogene homolog B (BRAF) and TERT promoter mutations, EGFR amplification, and Chromosome 7 and Chromosome 10 status.

The treatment received by patients was categorized into the following groups: (1) Full Stupp [1] (60 Gy/30 fractions with concurrent temozolomide and 6 cycles of adjuvant temozolomide), (2) Partial Stupp (stopped TMZ during RT or adjuvant phase), (3) Full Perry [21] (40 Gy/15 fractions with concurrent temozolomide and 6 cycles of adjuvant temozolomide), (4) Partial Perry (stopped TMZ during RT or adjuvant phase), (5) Short course RT only (40 Gy/15 fractions or 30 Gy/6 fractions), (6) Long RT only (60 Gy/30 fractions), (7) Other (chemo)radiotherapy, (8) Primary chemotherapy (no RT), and (9) No RT or chemo.

### 2.2. Imaging Review

All central nervous system CT and MR imaging for each patient was reviewed by a single consultant neuroradiologist with over 10 years of neuroimaging expertise (SC). This included any imaging before and after the imaging episode that demonstrated the TRA GBM. The final imaging reviewed constituted either the last imaging before death or, if still alive, the most recent imaging preceding the date of termination of data collection (31 March 2022). Archetypally, pre-operative imaging was performed at the presenting hospital, including the tertiary referral center. Consequently, pre-operative CT and MR imaging protocols varied with the imaging center. CT was typically performed using Siemens and GE Medical Systems machines with a range of 120–140 kV. Most commonly, MR images were acquired using a 1.5 T MRI scanner and consisted of T2 and FLAIR, diffusion-weighted imaging (DWI), T1-pre and post-gadolinium sequences (T1Gd), and a volumetric T1-weighted sequence post-gadolinium for surgical planning. No quantitative measurements of apparent diffusion coefficient (ADC) were analyzed; rather, the low signal on the ADC map of DWI was taken as a marker of reduced diffusivity, an interpretation that replicates clinical practice. Hounsfield Units of lesions on CT were calculated on the institutional picture archiving and communication system (PACS, Impax Version 6.5.3.3009, Agfa Healthcare, Mortsel, Belgium) using a region of interest (ROI, circle 2 points) with an area of 0.5 cm^3^.

The extent of peritumoral high T2 or FLAIR signal change was determined by a previously reported grading system [22]. This involved measuring radially from the tumor edge (T1Gd enhancing border if present; if no tumor enhancement, the neoplastic border was taken by the limit of the solid tumor bordering the imaging features of edema (the latter identified as low density on CT or T2 signal hyperintensity on MRI). The edema grade was as follows: Grade 0 = no edema, grade 1 = between 0 and 2 cm, and grade 2 = more than 2 cm from the tumor edge [22]. Enhancing tumor volume was estimated from orthogonal measurements in the axial and craniocaudal axes. These were measured on axial and coronal T1Gd images, or alternatively, using multiplanar reconstructions of a volumetric T1Gd sequence if available, using the institutional picture archiving and communication system (PACS, Impax Version 6.5.3.3009, Agfa Healthcare) with electronic calipers on a submillimeter (mm) scale. The axial image with the largest tumor was identified, and two maximum perpendicular dimensions were measured. Using reformatted sagittal or coronal images, the maximum dimension of the craniocaudal axis was measured. All three orthogonal measurements were multiplied and divided by 2 to estimate tumor volume [23]. When MR or T1Gd images were not acquired as part of the initial imaging assessment, estimates of tumor volume were made as outlined above, but with CT or T2-weighted imaging replacing T1Gd.

#### 2.2.1. Imaging Categorization

Patients were categorized into one of three groups that related to the typical radiological appearances of GBM (TRA GBM). TRA GBM was defined as a centrally necrotic, peripherally enhancing tumor with adjacent low CT density or high T2 MRI signal. The three groups were as follows:

1. Those with TRA GBM on imaging at presentation and subsequently confirmed with tissue diagnosis,
2. Those with a tissue diagnosis of GBM obtained when imaging showed TRA GBM but that had imaging preceding TRA GBM,
3. Those with a tissue diagnosis of GBM obtained when imaging showed non-typical GBM; that is, imaging at the time of tissue acquisition did not show a centrally necrotic, peripherally enhancing tumor with adjacent low CT density or high T2 MRI signal.

>1 locus of GBM was defined as more than one TRA GBM separated by normally appearing brain (i.e., no contiguous high T2 signal or enhancement connecting the lesions) [6]. Any molecular characteristics regarding >1 locus GBM refer to the first GBM resected in the patient unless otherwise specified.

#### 2.2.2. Incipient Image Patterns

Common imaging patterns across the groups were identified through image review by the same neuroradiologist looking at visual lesion characteristics including:

- CT density—low or high density relative to cerebral grey matter; Hounsfield units were also acquired as outlined above;
- T2 signal—relative to cerebral grey matter;
- The presence of reduced diffusivity on diffusion-weighted imaging, as outlined above;
- Enhancement pattern including none, solid (complete homogeneous enhancement), nodular (small foci of internal enhancement), complete peripheral and patchy peripheral (incomplete ring enhancement).

Patterns were reviewed by the researchers and agreed by group consensus.

### 2.3. Statistical Analysis

Any statistically significant differences between the TRA GBM and non-TRA GBM groups in terms of key demographic, oncological, and radiological features were sought. The Fisher exact test was used for categorical variables and one-way ANOVA for continuous variables. Both univariable and multivariable cox proportional hazards modeling was conducted to assess the association between the TRA GBM vs. non-TRA GBM groups and overall survival, allowing for adjustment of the other key clinical variables. EGFR amplification, chromosome 7, and chromosome 10 variables were not included in the multivariable model due to high levels of missing information. Statistical analyses were conducted in R Studio (v 2022.12.0+353) for MacOS X.

## 3. Results

Between 1 January 2014 and 31 March 2022, 555 patients had tissue-confirmed IDHwt GBM. These were divided into three groups based on the imaging features at tissue sampling:

- Non-typical GBM—patients who had tissue sampling and never showed TRA GBM, *n* = 20 (3.6%);
- TRA GBM at the time of tissue sampling with preceding imaging, *n* = 56 (10.1%);
- TRA GBM at the time of tissue sampling with no preceding imaging *n* = 479 (86.3%).

**Figure 1:**
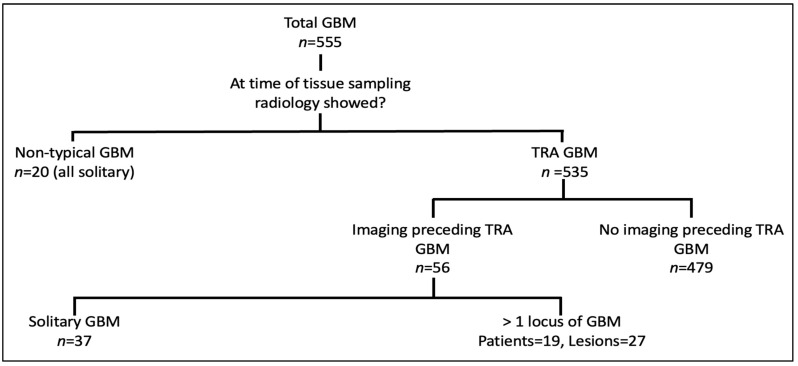
Derivation of lesions that did not show TRA GBM. Total number of lesions = 84; 57 solitary lesions and 27 with >1 locus. 20 of the 57 solitary lesions had tissue sampling when imaging never showed TRA GBM, so-called non-typical GBM.

All patients in the non-typical GBM group had a solitary lesion. The 56 patients that had TRA GBM at the time of tissue sampling and had preceding imaging were further divided into those with a solitary locus of GBM (*n* = 37) and those with >1 locus of GBM (*n* = 19). The 19 patients with >1 locus of GBM provided a total of 27 lesions that had imaging features that preceded TRA GBM (Figure 1).

In total, 84 lesions from 76 patients had imaging that either preceded TRA GBM or never showed TRA GBM. The patient demographics and lesion location for these 76 patients are summarized in Table 1.

### 3.1. Common Imaging Patterns

Common themes emerged from the review of the 84 lesions. It should be noted that not all patients had CT and MRI, owing to the retrospective nature of the study. The earliest imaging feature of the developing GBM was T2 hyperintensity +/− CT low density. A common finding was a lesion of CT hyperdensity, which on MRI appeared to correlate with (1) T2 iso-intensity (T2 iso), (2) reduced diffusivity on DWI, and (3) variable enhancement patterns including none, solid, nodular, and patchy peripheral. Lesional T2 iso/CT hyperdensity occurred later than T2 hyperintensity/CT low density. These imaging features will be further explored according to the subgroups: (a) non-typical GBM and (b) TRA GBM but with preceding imaging.

#### 3.1.1. Non-Typical GBM at Time of Tissue Sampling

**Figure 2:**
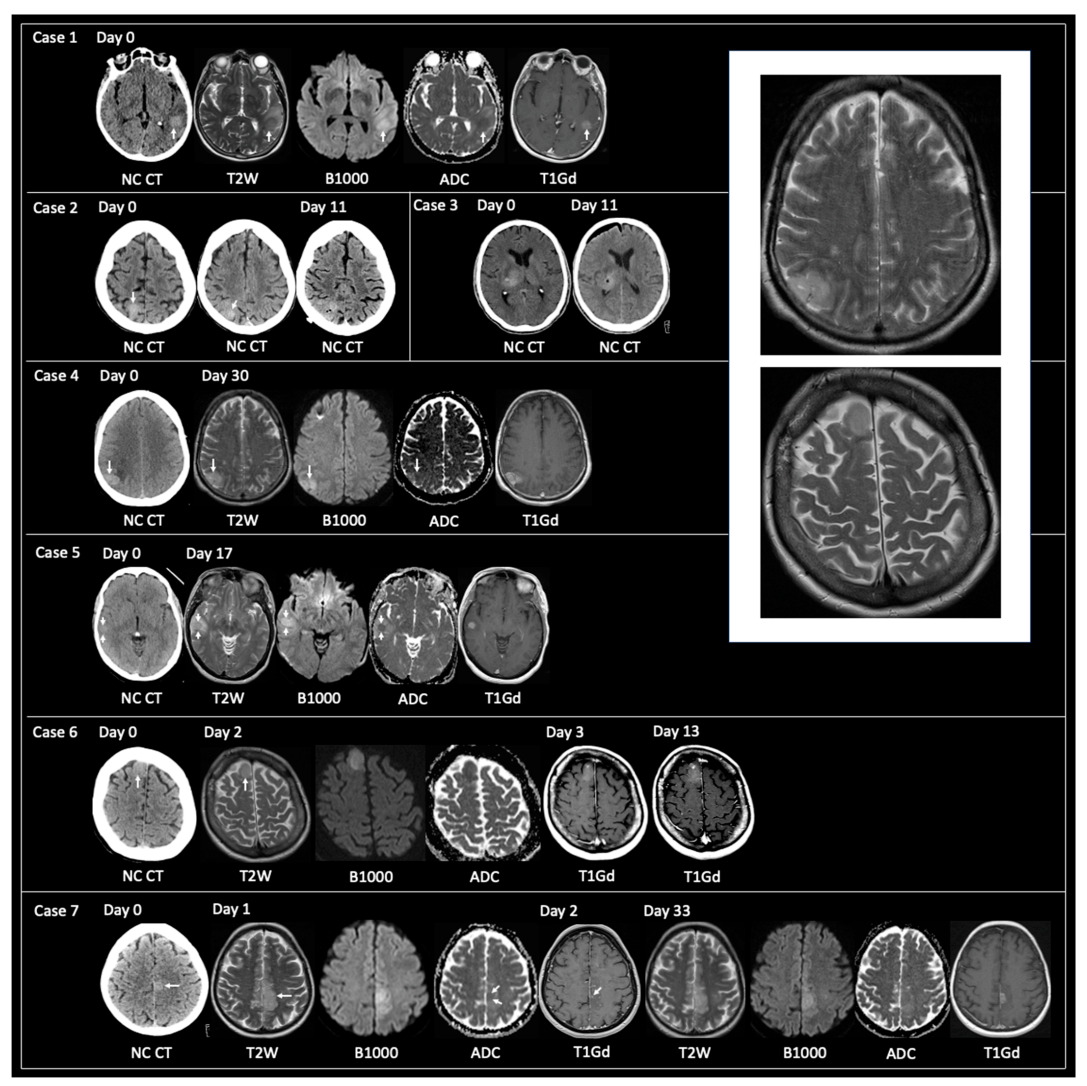
Examples of the imaging of non-typical GBM at the time of tissue sampling. **Case 1**: 72-year-old female presented with sudden-onset dysphasia. Unenhanced (non-intravenous contrast administration) CT (NC CT) showed a hyperdense lesion in the posterior aspect of the left superior temporal gyrus (arrow). MRI obtained on the same day showed that the lesion had corresponding T2 iso, solid enhancement, and reduced diffusivity (high B1000, low apparent diffusion coefficient (ADC)). **Case 2**: 74-year-old female was referred for memory impairment. Unenhanced CT showed a hyperdense lesion in the right superior parietal lobule (two contiguous CT slices shown). No MRI was obtained due to the patient having an incompatible permanent pacemaker. Unenhanced CT at day 11 showed post-biopsy appearances with small volume pneumocephalus within the hyperdense lesion. **Case 3**: 62-year-old male presented with left-sided weakness. Unenhanced CT showed a hyperdense lesion in the right thalamus (left image). Post-biopsy unenhanced CT (right image) with frontal subdural pneumocephalus and small focus of pneumocephalus at the biopsy site. MRI was contraindicated. **Case 4**: 50-year-old male presented with a seizure. Unenhanced CT revealed a hyperdense lesion in the right inferior parietal lobule. MRI at day 30 showed corresponding T2 iso, reduced diffusivity, and solid enhancement. **Case 5**: 56-year-old female presented with a seizure. Unenhanced CT at day 0 showed a hyperdense lesion in the right temporal lobe. MRI on day 17 showed that the lesion was T2 iso, had internal reduced diffusivity with corresponding solid enhancement. **Case 6**: 52-year-old female presented with a seizure. Hyperdense lesion in the anterior aspect of the right superior frontal gyrus on unenhanced CT at day 0. This had corresponding T2 iso and reduced diffusivity on MRI obtained on day 2. T1-weighted post-gadolinium (T1Gd) imaging obtained on day 3 and day 13 showed solid tumoral enhancement increasing in size over the 10-day interval. **Case 7**: 56-year-old male presented with a seizure. Unenhanced CT demonstrated a hyperdense lesion in the left paracentral lobule with surrounding low attenuation in left frontal and parietal lobes. MRI on day 1 revealed a T2 iso infiltrative lesion with a focus of reduced diffusivity and tiny dot of solid enhancement in the area of hyperdensity shown on the initial CT. MRI at day 33 showed a solid tumor (no necrosis) with a greater volume of solid enhancement at the site of prior CT hyperdensity. The Box in the top right of the image shows two enlarged examples of GBM where the tumor signal on T2-weighted imaging is isointense (T2 iso) to grey matter (taken from **Cases 4 and 6**). NC CT—non-contrast CT, T2W—T2-weighted, B1000—DWI, ADC—apparent diffusion coefficient map of DWI, T1Gd—T1-weighted post-gadolinium.

20 patients had a tissue diagnosis of GBM when imaging showed non-typical GBM. All patients had either a CT hyperdense or T2 iso-lesion: 3 of 20 patients had CT only, 7 of 20 had MRI only, and 10 of 20 had an initial CT followed by MRI with a mean duration between the imaging studies of 10 days (range 0–30). All 10 patients with CT and MRI showed a CT hyperdense lesion that corresponded to T2 iso. All 17 patients that had MRI had T1Gd and the enhancement patterns of the 17 lesions were as follows: none = 1, solid = 6, and nodular = 10. Here, 16 of the 17 patients that had MRI had accompanying DWI, and all 16 lesions showed reduced diffusivity. Examples of lesions with non-typical GBM are shown in Figure 2—note case 7 that shows an intratumoral focus of CT hyperdensity that progresses at a faster rate than the rest of the tumor.

Radiologist impression for the CT hyperdensity at the time of the initial CT was as follows: metastasis or primary tumor (*n* = 5), primary CNS neoplasm (*n* = 2), infarct (*n* = 2), indeterminate (*n* = 2), hemorrhagic infarct (*n* = 1), and lymphoma (*n* = 1). Radiologist impression for the T2 iso at the time of the initial MRI was as follows: glioma (*n* = 3), primary CNS neoplasm (*n* = 2), metastasis or primary tumor (*n* = 1), and encephalitis (*n* = 1).

#### 3.1.2. TRA GBM with Preceding Imaging

The 56 patients with 64 lesions had imaging that preceded TRA GBM. 40/64 lesions had CT as the initial investigation and in 38/40 (88%) cases, the lesion was hyperdense; 2/40 (12%) lesions were hypodense. 24/64 lesions had MRI as the initial investigation, and 16/24 (67%) lesions were initially T2 hyperintense; 8 (33%) lesions showed T2 iso. 31/38 hyperdense lesions had short-interval MRI (mean time from CT to MRI = 9 days (range 0–63)). All 31 hyperdense lesions showed corresponding T2 iso. 28/31 lesions had DWI available, and all lesions showed reduced diffusivity. 23/31 lesions had T1Gd available, and the following enhancement patterns were observed: no enhancement = 6 (26%), solid = 5 (22%), nodular = 9 (39%), and patchy peripheral = 3 (13%).

**Figure 3:**
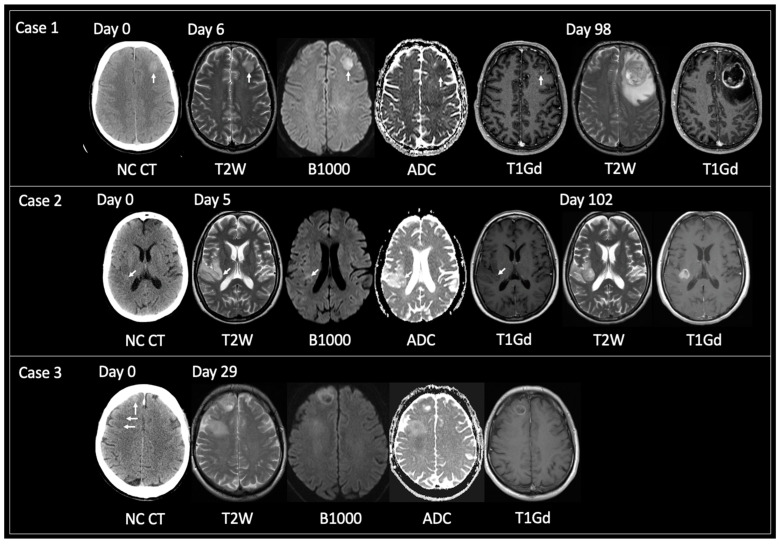
Examples of solitary lesions with TRA GBM at tissue diagnosis that had preceding imaging. **Case 1**: 56-year-old male presented with seizures. Unenhanced CT on day 0 showed a hyperdense lesion in the left frontal lobe. This had corresponding T2 iso signal with reduced diffusivity and central nodular enhancement on MRI day 6. TRA GBM followed on MRI day 98. **Case 2***:* 53-year-old female presented with several days of intermittent left-sided anesthesia. Initial unenhanced CT day 0 showed a focus of hyperdensity within the posterior aspect of the right insula (arrow) with adjacent cortical low density. MRI day 5 revealed an infiltrative glioma within the right inferior parietal lobule and right insular with the focus of prior CT hyperdensity corresponding to T2 iso signal, reduced diffusivity and containing a tiny dot of enhancement (arrows). MRI day 102 showed TRA GBM at the site of the prior CT hyperdense focus but relative stability of the rest of the non-enhancing tumor. **Case 3**: 63-year-old male presented with seizures. Unenhanced CT day 0 revealed two abnormal areas: a hyperdense focus anteriorly in the right superior frontal gyrus (vertical arrow) and subcortical hypodensity in the right middle frontal gyrus (horizontal arrows). These foci were linked by hypodensity (not shown) correlating with one diffuse tumor. MRI day 29 demonstrated interval growth of the previously hyperdense lesion, showing TRA GBM with enhancing periphery and central necrosis. Note how the peripheral tumoral tissue shows T2 iso solid signal with reduced diffusivity and enhancement. MRI day 29 also showed progression of the previously low-density lesion but not to TRA GBM. This lesion was shown to infiltrate into the deep white matter of the right centrum semiovale, contain areas of reduced diffusivity but no enhancement. TRA GBM—typical radiological appearance of glioblastoma, NC CT—non-contrast CT, T2W—T2-weighted, B1000—DWI, ADC—apparent diffusion coefficient map of DWI, T1Gd—T1-weighted post-gadolinium.

Mean time from CT hyperdensity to TRA GBM (*n* = 38) = 74 days (range 7–158); solitary lesions only (*n* = 32) = 69 days (7–158); >1 locus only (*n* = 6) = 95 days (46–147). Figure 3 displays examples of solitary lesions with TRA GBM at the time of tissue sampling with preceding imaging. Note cases 2 and 3 that show intratumoral foci of CT hyperdensity that progress at a faster rate than the rest of the tumor.

**Figure 4:**
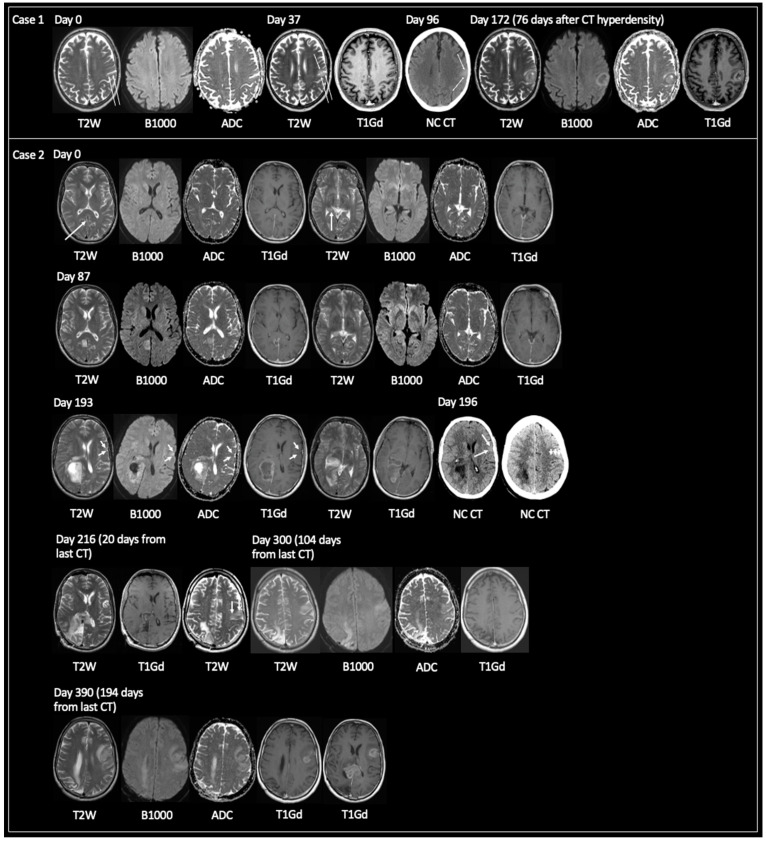
Examples of early GBM progressing through T2 hyperintensity to T2 iso and CT hyperdensity prior to showing TRA GBM. **Case 1**: 68-year-old male presented with dysphasia at day 96 leading to unenhanced CT investigation, which showed a solitary hyperdense lesion in the anterolateral aspect of the left parietal lobe, bordering the left supramarginal gyrus. MRI at day 172 showed TRA GBM with central necrosis and peripheral enhancement. MRI at day 0 obtained for an 8-week history of left-sided hearing loss revealed subtle cortical expansion at the site of future GBM (arrows) with no reduced diffusivity. On MRI day 37 the cortical expansion was more apparent showing relative growth to day 0. **Case 2**: 59-year-old male with progressively worsening headaches. MRI day 0 showed small foci of T2 signal hyperintensity in the cortex of the right precuneus (small arrow) and within the posterior aspect of the right parahippocampal gyrus (long vertical arrow). Neither the focus of T2 signal hyperintensity showed reduced diffusivity nor enhancement. Note the smooth dural thickening and enhancement on the T1Gd images. MRI day 87 showed interval growth of the lesion in the right precuneus with some central necrosis and peripheral enhancement. The lesion in the right parahippocampal gyrus had also grown relative to day 0 and continued to show facilitated diffusion and nonenhancement. The working diagnosis at the time of day 0 and day 87 was granulomatosis with polyangiitis, as the patient was ANCA positive and had no other known cause for dural enhancement. MRI day 193 showed growth at both lesion sites with TRA GBM. Note the new small ovoid focus of cortical T2 iso in the left inferior frontal gyrus (arrows), showing concomitant reduced diffusivity but no enhancement. An unenhanced CT obtained 3 days later, on day 196, showed corresponding CT hyperdensity in the lesion in the left inferior frontal gyrus and this subsequently developed into TRA GBM on MRI day 216. Note also the cortical T2 iso-lesion laterally in the posterior aspect of the left frontal lobe on MRI day 216 (3 vertical arrows), not clearly represented on CT day 196 (3 vertical arrows). This subsequently progressed to TRA GBM on MRI day 390 after showing growth and T2 iso signal on MRI day 300 with concomitant high B1000 signal but without enhancement (MRI day 300 was obtained 2 week after the patient’s third cycle of adjuvant TMZ—no RT was given as the patient declined whole brain RT, which was deemed the only valid RT option). Additionally, note TRA GBM within the splenium of the corpus callosum on MRI day 390 representing local progression of the original lesion (3 weeks following the patients sixth cycle of adjuvant TMZ). TRA GBM—typical radiological appearance of glioblastoma, NC CT—non-contrast CT, C+ CT—intravenous contrast-enhanced CT, T2W—T2-weighted, B1000—DWI, ADC—apparent diffusion coefficient map of DWI, T1Gd—T1-weighted post-gadolinium, RT—radiotherapy, TMZ—temozolomide, ANCA—antineutrophilic cytoplasmic antibody.

For 18/84 lesions, the earliest imaging features of GBM were T2 hyperintensity (*n* = 16) and CT hypodensity (*n* = 2). 13/16 T2 hyperintense lesions had accompanying DWI, and no lesion showed reduced diffusivity. 11/16 had T1Gd: 10 had no enhancement; 1 had a tiny dot of nodular enhancement. 7/18 lesions later showed T2 iso. Mean time from T2 hyperintensity/CT low density (*n* = 18) to TRA GBM = 173 days (43–537); solitary lesions only (*n* = 3) = 140 days (43–267); >1 locus only (*n* = 15) = 179 days (69–537). Figure 4 displays examples of lesions passing through phases of T2 hyperintensity to T2 iso/CT hyperdensity to TRA GBM.

Mean Hounsfield Units across all CT hyperdense lesions (*n* = 51) were 46 HU (range 33–59), for hyperdense non-TRA GBM only (*n* = 13) were 46 HU (37–59), and for hyperdense lesions in the group with imaging that preceded TRA (*n* = 38) were 46 HU (33–56). The Hounsfield Units of cerebral white matter and grey matter are approximately 25 HU and 35 HU, respectively. The standard deviation of HU values is usually in the ± 10–20% range [24].

Table 2 compares patient and lesion characteristics between three groups, all with solitary lesions: (1) non-typical GBM at the time of tissue sampling; (2) TRA GBM at the time of tissue sampling with preceding imaging; and (3) TRA GBM at the time of tissue sampling with no prior imaging. Compared to patients who had tissue sampling for TRA GBM, the non-typical GBM cohort had statistically significant smaller tumor volume and less surrounding edema. There was no statistically significant difference between the groups for age, sex, overall survival, or molecular characteristics, except for fewer Chromosome 7 polysomies in the non-TRA GBM group.

## 4. Discussion

The purpose of this study was to investigate whether developing GBM displays a spectrum of imaging changes that are detectable on routine clinical imaging. Results suggest that GBM begins as an abnormal focus of high T2 signal intensity predominantly, but not exclusively, in the cerebral cortex. As the tumor grows, this signal changes to T2 iso. This stage in tumor development is associated with CT hyperdensity, reduced diffusivity, no or little surrounding high T2 signal, and variable enhancement, the latter ranging from none, nodular, solid to patchy peripheral. The solid-appearing T2 iso signal may then be replaced with a focus or foci of necrosis, leading to the TRA GBM of central necrosis and peripheral enhancement with surrounding high T2 signal (for example, Figure 3, Case 1). This pattern of growth was evidenced for solitary and multiple loci of GBM, for GBM in cortical and white matter locations, and in patients who were receiving or had received treatment.

Speculatively, the imaging features and underlying pathophysiology may broadly correspond as follows: (1) initial high T2 signal represents early tumor growth and non-cytotoxic edema; (2) CT hyperdensity, T2 iso, and reduced diffusivity symbolize a solid tumor mass of rapidly proliferating cellular hyperdensity [25,26]; (3) enhancement corresponds to vascular endothelial proliferation and loss of the normal integrity of the blood-brain barrier (BBB) [27]; (4) further changes to the tumor environment lead to cell death and intratumoral necrosis [28]; and (5) peritumoral high T2 signal relates to tumor invasion and edema [4].

GBM as a CT hyperdense lesion has been reported previously [20,29]. Over a 10-year period, Ceravolo et al. [20] found 13/430 (3%) patients with imaging features prior to TRA GBM; mean age 63 years (48–86), M:F = 9:4. One patient had two loci of GBM; 14 lesions in total. Lesion location comprised: cortical *n* = 6; cortical/subcortical *n*= 7; white matter *n* = 1. 7/14 had a CT hyperdense lesion. 3/14 had tissue sampling prior to TRA GBM and two of these showed CT hyperdensity. The mean time from ‘early GBM’ (as they are referred) to TRA GBM was 3 months (4 days to 12 months). The present study is substantially larger than that offered previously. It also provides Hounsfield Units (not previously offered) and is more comprehensive in its coverage of volumetric and molecular tumoral data. The temporal duration of TRA GBM is similar to that of Ceravolo et al. Unlike prior studies, the current research also highlights the importance of early T2 hyperintensity moving to T2 iso and/or CT hyperdensity for the detection of the developing GBM. Moreover, the current research provides examples where tumor foci showing CT hyperdensity and/or T2 iso within a larger lesion grow faster than the rest of the tumor (for example, Figure 2, Case 7 and Figure 3, Case 2). Hypothetically, this may be explained by intratumoral heterogeneity—a concept referring to the unique phenotypic, genetic, and functional differences arising across the landscape of an individual tumor [4,30,31]. This heterogeneity enables distinct cancer cell populations to behave and respond differently to environmental factors, including in response to therapeutic strategies [4,30,31]. Future studies may wish to further explore image-guided multi-regional tissue sampling to better understand the molecular determinants of rapid proliferation.

The majority of the 84 GBM lesions in this study originated from the cerebral cortex. The cortical origin of GBM is consistent with a murine model that used fluorescent labeling with single-cell resolution to track tumorigenesis; this model provided strong evidence for GBM formation in grey matter [32]. Most patients in this cohort presented with seizures, presumably secondary to neoplastic infiltration into the cortex. Other patients present with focal neurologic deficits, with the tumor developing in eloquent areas of the brain [33]. However, this present research has also shown that cortical origin is not exclusive to GBM. Furthermore, GBMs have also been reported to originate and recur in the subventricular zone, and it is possible that tumors arising in the cortex are due to the secondary outward migration of abnormal brain tumor cells [34,35], providing an explanation for lesions that develop in deep and subcortical white matter. Indeed, an interesting observation in this current study was the development of GBM at the olfactory bulb in four patients that had received prior radiotherapy for an earlier remote GBM. Human GBM cells injected into immunodeficient nude mice have been shown to preferentially migrate to olfactory bulbs [36]. Additionally, olfactory bulbs have been implicated as a radioresistant niche for GBM cells [37]. Conversely, olfactory bulbs are also a rich source of neural stem cells [38], and the generation of a new, de novo GBM from local cellular mutations rather than as a result of cell migration and proliferation from a primary lesion cannot be discounted.

Although no statistically significant difference in OS was found between the TRA GBM and non-TRA GBM groups, the number of patients in the latter cohort is small. The literature supports improved prognosis with earlier GBM detection. Smaller tumor volume [6], maximal surgical resection [39,40,41,42,43] and preoperative MR imaging displaying little or no necrosis, little tumor enhancement, and a lesser degree of peritumoral edema are associated with better prognosis [44]. These were the imaging features found in the non-TRA GBM group and the early stages of the group with imaging preceding TRA GBM. Future research using machine learning techniques could evaluate the possibility of non-typical GBM detection, not only for earlier detection but also for the use of radiotherapy planning and surveillance to determine those tumor niches that are prone to rapid proliferation. Such research would align with the current move toward radiogenomics and precision medicine [45].

This study has several limitations. It is retrospective, from a single center, and used only a single experienced neuroradiologist as an observer. A further limitation is the unavailability of population-level imaging to assess the true prevalence of non-typical GBM, as not all patients with suspected GBM were included—given that some patients do not undergo tissue diagnosis due to poor performance status. Additionally, it is also acknowledged that in patients with >1 locus of GBM, only one locus underwent tissue sampling. It cannot be determined, therefore, whether separate loci represented de novo tumors or resulted from the distant spread of the co-existing lesion. Finally, in a subset of patients, it cannot be discounted that lesions may have shown enhancement earlier if intravenous contrast was administered at an earlier imaging episode.

## 5. Conclusions

Developing GBM appears to display a spectrum of imaging features moving through phases of T2 hyperintensity to CT hyperdensity, T2-iso, reduced diffusivity, and variable enhancement before showing TRA GBM. The mean time of progression to TRA GBM from T2 hyperintensity and from CT hyperdensity is 140 days and 69 days, respectively. A CT cortical/subcortical hyperdense lesion that shows concomitant T2 iso and reduced diffusivity, irrespective of the presence or absence of enhancement, should raise red flags for non-typical GBM and trigger urgent diagnosis and treatment. Future research correlating this imaging spectrum with pathophysiology may provide insight into GBM growth, particularly by examining for possible differences that may exist in molecular biology between the features described in this cohort and those lesions with typical radiological appearances of GBM.

## Figures and Tables

**Table 1:** Characteristics of patients and lesions with imaging that preceded or never showed TRA GBM. 76 patients, 84 lesions. * Presenting complaint denotes the clinical reason for the initial imaging which showed an abnormality prior to TRA GBM and is inclusive of solitary lesions only, as it was clinically difficult to assign a presentation to a particular lesion in those patients with >1 locus of GBM and many patients with >1 locus of GBM had TRA GBM at the time of presentation. ** All olfactory groove lesions were secondary loci of GBM.

Parameter	Quantity
Mean age (range, years)	
Entire cohort (*n* = 76)	61 (18–81)
Solitary (*n* = 57)	61 (18–81)
>1 locus (*n* = 19)	60 (42–80)
M:F	
Entire cohort (*n* = 76)	45:31
Solitary (*n* = 57)	30:27
>1 locus (*n* = 19)	15:04
Overall survival (months, range)	
Entire cohort (*n* = 55 diseased at censor date)	15 (1–57)
Solitary (*n* = 37)	16 (1–57)
>1 locus (*n* = 18)	13 (3–26)
Presenting complaint (%) *	
Seizure	36 (63.0)
Dysphasia	8 (14.0)
Unilateral weakness or sensory change	6 (10.5)
Memory impairment	2 (3.5)
Homonymous hemianopia	3 (5.0)
Change in behavior	1 (2.0)
Atremulous Parkinson’s	1 (2.0)
Lesion location *n* = 84 (%)	
R:L cerebral hemisphere	43:41
Grey–white matter location	
Cortical	60 (71.5)
Cortical/subcortical	10 (12.0)
Subcortical	10 (12.0)
Deep white matter	3 (3.5)
Deep grey matter	1 (1.0)
Cerebral location (%)	
Temporal	27 (32.0)
Frontal	24 (28.5)
Parietal	10 (12.0)
Occipital	8 (9.5)
Olfactory grove	4 (5.0) **
Paracentral lobule	2 (2.5)
Insula	2 (2.5)
Parieto–temporal	2 (2.5)
Parieto–occipital	2 (2.5)
Subcentral gyrus	1 (1.0)
Centrum semiovale	1 (1.0)
Thalamus	1 (1.0)

**Table 2:** Comparison between three groups, all with solitary lesions: (1) non-typical GBM at the time of tissue sampling; (2) TRA GBM at time of tissue sampling with preceding imaging; and (3) TRA GBM at time of tissue sampling with no preceding imaging.

Variableat Time of Tissue Sampling	Non-Typical GBM (*n* = 20)	TRA GBM but with Imaging Preceding (*n* = 37)	TRA GBM with No Imaging Preceding (*n* = 479)	Statistical Significance (TRA GBM vs. Non-Typical GBM)
Mean age, years (range)	61 (27–81)	61 (18–77)	61 (25–86)	*p* = 0.94
M:F proportion	9:11	21:16	307:172	*p* = 0.13
OS, months (range) *	17 (2–49)	16 (1–57)	10 (0–80)	0.67
Oedema grade with initial lesion (%)				Overall *p* < 0.001
No edema	15 (75.0)	25 (68.0)	10 (2.0)	<0.001
0–2 cm edema	5 (25.0)	12 (32.0)	125 (26.0)	1
>2 cm edema	0 (0.0)	0 (0.0)	344 (72.0)	<0.001
Mean tumor volume with initial lesion (cm^3^)	5.5 (range 0.5–21.9)	4.1 (0.2–18.8)	34.4 (0.1–185.9)	*p* < 0.001
Surgical resection: biopsy	9:11	30:7	336:143	*p* = 0.09
% BRAF wild type (fraction of samples with a known result)	100% (14/14)	95% (21/22)	98% (287/294)	*p* = 1
TERT mutation (fraction of samples with a known result)	93% (13/14)	87% (20/23)	89% (259/291)	*p* = 0.67
EGFR amplified (fraction of samples with a known result)	67% (8/12)	60% (9/15)	42% (30/72)	*p* = 0.12
Chromosome 7 polysomy (fraction of samples with a known result)	25% (1/4)	60% (3/5)	85% (22/26)	*p* = 0.03
Chromosome 10 monosomy (fraction of samples with a known result)	33% (1/3)	100% (5/5)	83% (19/23)	*p* = 0.12
MGMT hypermethylation (fraction of samples with a known result)	59% (10/17)	38% (12/32)	38% (155/403)	*p* = 0.08
Treatment received				Overall *p* = 0.94
Full Stupp	5 (25.0)	15 (40.5)	87 (18.0)	*p* = 0.56
Partial Stupp	7 (35.0)	7 (18.95)	126 (26.0)	*p* = 0.44
Full Perry	0 (0.0)	3 (8.1)	9 (2.0)	*p* = 1
Partial Perry	0 (0.0)	3 (8.1)	23 (5.0)	*p* = 1
Short course RT only	4 (20.0)	7 (18.95)	103 (22.0)	*p* = 1
Long RT only	0 (0.0)	0 (0.0)	21 (4.0)	*p* = 1
Other (chemo)radiotherapy	0 (0.0)	1 (2.7)	17 (4.0)	*p* = 1
Primary chemotherapy (no RT)	1 (5.0)	0 (0.0)	20 (4.0)	*p* = 0.42
No RT or chemo	3 (15.0)	1 (2.7)	73 (15.0)	*p* = 0.55

* At the censor date, 11/20, 37/37 and 372/479 patients of the non-typical GBM, TRA GBM with prior imaging & TRA GBM with no prior imaging, respectively, were deceased. Survival difference for non-TRA GBM vs. TRA GBM shown is for multivariable model = Hazard ratio (non-TRA) = 1.28 (95% CI 0.41–4.05, *p* = 0.67); univariable model = Hazard ratio (non-TRA) = 0.66 (95% CI 0.36–1.19, *p* = 0.17). BRAF—v-Raf murine sarcoma viral oncogene homolog B, TERT—telomerase reverse transcriptase, EGFR—epidermal growth factor receptor, MGMT—O6-methylguanine-DNA methyltransferase. RT—radiotherapy. Gy—Gray (International System radiation dose; 1 Gy = 1 Joule/kilogram). Treatment: Full Stupp [1] (60 Gy/30 fractions with concurrent temozolomide and 6 cycles of adjuvant temozolomide), Partial Stupp (stopped TMZ during RT or during adjuvant phase), Full Perry [21] (40 Gy/15 fractions with concurrent temozolomide and 6 cycles of adjuvant temozolomide), Partial Perry (stopped TMZ during RT or during adjuvant phase), Short course RT only (40 Gy/15 fractions or 30 Gy/6 fractions), Long RT only (60 Gy/30 fractions), Other (chemo) radiotherapy, Primary chemotherapy (no RT), and No RT or chemotherapy.

## Data Availability

Restrictions apply to the availability of these data due to local institutional information governance policy.
